# Oxidative Stress in Microbial Diseases: Pathogen, Host, and Therapeutics

**DOI:** 10.1155/2019/8159562

**Published:** 2019-01-10

**Authors:** Rômulo Dias Novaes, Antônio Lúcio Teixeira, Aline Silva de Miranda

**Affiliations:** ^1^Institute of Biomedical Sciences, Federal University of Alfenas, Alfenas, 37130-001 Minas Gerais, Brazil; ^2^Department of Structural Biology, Federal University of Alfenas, Alfenas, 37130-001 Minas Gerais, Brazil; ^3^Neuropsychiatry Program, Department of Psychiatry & Behavioral Sciences, McGovern Medical School, University of Texas Health Science Center at Houston, Houston, TX, USA; ^4^Interdisciplinary Laboratory of Medical Investigation, School of Medicine, Federal University of Minas Gerais, Belo Horizonte, MG, Brazil; ^5^Laboratory of Neurobiology, Institute of Biological Sciences, Department of Morphology, Federal University of Minas Gerais, Belo Horizonte, MG, Brazil

As central components of the “respiratory burst” in activated leukocytes, the reactive oxygen species (ROS) and reactive nitrogen species (RNS) play an essential role in the host immune defenses against pathogens [1, 2]. However, based on their nonspecific and highly reactive nature, radical and nonradical products of redox metabolism (e.g., O2^·-^, OH^·-^, ONOO^·-^, HClO, H_2_O_2_, and NO) may be potentially harmful to host cells [1, 2]. Apart from virulence factors expressed by pathogenic microorganisms (e.g., microbial cytotoxins), the excessive production of ROS and RNS by activated immune cells creates a highly cytotoxic milieu that contributes to the direct damage of target organs in the context of unbalanced inflammatory responses [1–4].

Despite their molecular “promiscuity,” ROS and RNS belong to a set of molecules that establish the complex interface between susceptibility versus resistance to infectious agents [1–3]. Accordingly, the modulation of the host's redox system as an antimicrobial therapeutic strategy is challenging. The upregulation of ROS and RNS production may increase cytotoxicity and organ damage [3], while the downregulation of these molecules may favor the survival and spread of pathogenic microorganisms, leading to opposite scenarios with a similar endpoint, including higher mortality of the host [3, 4].

Emerging evidence suggests that the modulation of the redox metabolism of different microorganisms is more rational (i.e., bacterium, fungi, protozoan, and virus) as a therapeutic strategy, instead of manipulating the redox system of vertebrate hosts. The redox metabolism in microorganisms is based on a rudimentary enzymatic process compared with the complex and multiscale enzymatic (e.g., superoxide dismutase, catalase, glutathione reductase, and glutathione S-transferase) and nonenzymatic (e.g., vitamins C and E, *β*-carotene, and uric acid) pathways broadly distributed in fluids and tissues of vertebrate hosts [4, 5]. As several antioxidant enzymes of pathogenic microorganisms are not expressed in eukaryotic hosts, these enzymes might represent potentially promising molecular targets for novel drug design [6, 7].

Antioxidant systems seem to play a crucial role in maintaining the morphological and functional integrity of all microorganisms. Accordingly, disruptors of redox balance (e.g., inductors of oxidative stress or inhibitors of antioxidant molecules) have been proposed as candidates to new antimicrobial drugs [4, 6]. Ideally, modulators of redox systems should be able to influence microbial metabolism with minimal or no interference on the host antioxidant defenses [6, 7]. Interestingly, several drugs developed for different conditions (e.g., anti-inflammatory, antineoplastic, antidepressant, anxiolytic, and antipsychotic) also exhibit antimicrobial properties [8, 9]. In many cases, these properties are based on the disruption of microbial redox metabolism, a pharmacological effect that opens new venues for drug repurposing and development of new strategies for the treatment of infectious diseases [8, 9].

This special issue gathers a set of eight studies in an interdisciplinary platform that addresses the subcellular, cellular, and molecular bases of the metabolism redox associated to infectious diseases. This special issue also highlights the continuing effort to understand the redox systems from microbial metabolism, host-pathogen interaction, and treatment of several infectious diseases. It contains eight papers describing different microbial species and pathological conditions, which are briefly commented below.

Considering an approach based on drug repositioning, A. A. S. Mendonça et al. used a multiscale framework to investigate the anti-*Trypanosoma cruzi* properties of a heterogeneous class of drugs with potential to inhibit trypanothione reductase (TR) in trypanosomatids. While molecular docking corroborated the affinity and inhibition of TR by drugs such as clomipramine and thioridazine, these molecules were also effective in attenuating *T. cruzi* infection *in vivo*. As in silico molecular affinity studies were unable to explain *T. cruzi* death *in vitro* and the antiparasitic potential of these drugs *in vivo*, the authors proposed that the anti-*T. cruzi* effects of these drugs are not restricted to TR inhibition. They also suggested that TR inhibitors might be interesting candidates for antitrypanosomal chemotherapy, although their therapeutic potential needs to be further explored.

C. Lee reported that virus-induced oxidative stress plays a critical role in the pathogenesis of several viral diseases. During viral infections, host cells seem to activate an important mechanism of ROS cytoprotection mediated by the 2p45-related factor 2 (Nrf2) pathway. Additionally, the author indicated that a group of clinically relevant viruses is able to subvert the Nrf2 pathway, modulating host antioxidant response and allowing infection progression. In addition to viruses that positively or negatively modulate the Nrf2 pathway, specific strategies based on Nrf2 manipulations with potential to suppress viral replication and virus-induced oxidative damage were also analyzed.

M. D. Shastri et al. described the emerging problem related to multidrug-resistant tuberculosis and the challenges associated to antibacterial chemotherapy. The authors reviewed the relationship between lung oxidative stress and its effects on the activation of specific antibiotics. They also discussed strategies used by drug-susceptible and drug-resistant *M. tuberculosis* subtypes to survive and establish the infection. From a comprehensive background, new therapies based on standard antibiotics combined with adjuvant agents were proposed to overcome *M. tuberculosis* resistance to host prooxidant mechanisms.

Y. Lin et al. pointed out *Propionibacterium acnes* as a relevant agent associated with intervertebral disc degeneration (IVDD). In humans infected with *P. acnes*, cases of more severe IVDD were associated with increased iNOS/NO and COX-2/PGE2 activity. By employing a rat model, the authors showed that the upregulation of iNOS/NO and COX-2/PGE2 was essential to the occurrence of *P. acnes*-induced IVDD. Importantly, the inhibition of these molecules was associated with the restoration of aggrecan and collagen II expression, with beneficial effects in attenuating IVDD. In this study, a strong involvement of *P. acnes* in IVDD as well as the underlying pathogenic mechanisms associated with proinflammatory and prooxidant events was shown.

D. M. S. Pereira et al. investigated the contribution of transient receptor potential canonical 5 (TRPC5) and transient receptor potential canonical 4 (TRPC4) to bacterial thioredoxin-induced responses in lipopolysaccharide- (LPS-) injected mice. They corroborated the evidence that *E. coli*-derived thioredoxin is a virulence factor that potentiates mortality in LPS-injected mice. By using TRPC5 wide-type (TRPC5^+/+^) and knockout (TRPC5^−/−^) mice, as well as TRPC4/TRPC5 antagonists, the study clearly showed that the blockage of these receptors modulates the systemic inflammatory response and increases the mortality in thioredoxin-treated LPS mice. Thus, TRPC4/TRPC5 seems to modulate *E. coli*-derived thioredoxin virulence by exerting a pathophysiological role similar to infections caused by other bacteria species.

C. Xie et al. investigated the role of ROS in the pathogenesis of *Helicobacter pylori*-related disease and the protective effect of N-acetyl-cysteine, an antioxidant compound. From *in vitro* and *in vivo* models, the authors showed that *H. pylori* infection increased ROS production and DNA damage in gastric epithelial cells, a process mediated by PI3K/Akt pathway activation. Treatment with N-acetyl-cysteine decreased ROS levels and DNA damage in gastric epithelial cells and gastric mucosa, with a limited influence on the gastric mucosa pathological score of *H. pylori*-infected Balb/c mice. Considering these findings, the authors suggest that N-acetyl-cysteine may prevent the DNA damage during *H. pylori*-related gastric diseases by attenuating ROS levels.

Admitting the role of autophagy machinery in the defense against microorganisms, Z. Duan et al. investigated the influence of *Candida albicans* on autophagic flux in macrophages. Phagocytosis of *C. albicans* decreases autophagic flux, but induces LC3-associated phagocytosis (LAP) and ROS production in a MTOR-independent manner. Altogether, these findings indicate that the occupation of microtubule-associated protein 1 light chain 3 (LC3) by recruiting engulfed *C. albicans* might contribute to the inhibition of autophagic flux. By employing an *in vitro* approach, the authors showed a coordinated machinery between canonical autophagy and LAP in macrophages, which is recruited from *C. albicans* challenge.

S. Zhang et al. explore the effects of xylanase and fermented polysaccharide of *Hericium caputmedusae* (FPHC) on pathogenic infection in broilers. Although broilers have been affected by bacterial pathogens, the dietary supplementation with xylanase and FPHC improved anti-inflammatory and antioxidant capacity, with a positive impact on intestinal microbiota. From an agroindustrial perspective, the supplementation with xylanase and FPHC can be a potential alternative to increase poultry productivity.

We hope that the readers of this special issue will find these findings interesting and useful to advance the understanding of such a complex and multifaceted theme, making provocative the update of this interesting subject.
